# Retraction Note: METTL13 is downregulated in bladder carcinoma and suppresses cell proliferation, migration and invasion

**DOI:** 10.1038/s41598-023-39010-y

**Published:** 2023-07-24

**Authors:** Zhe Zhang, Guojun Zhang, Chuize Kong, Bo Zhan, Xiao Dong, Xiaojun Man

**Affiliations:** 1 Department of Urology, the First Hospital of China Medical University, Shenyang, 110001 China; 2 Department of Hematology, Shengjing Hospital of China Medical University, Shenyang City, 110022 China

Retraction of: *Scientific Reports* https://doi.org/10.1038/srep19261, published online 14 January 2016

The Editors have retracted this article.

Initial concerns were raised about the fact that the tumours in Fig. 6A appear to have been pasted into the image individually, as well as a partial overlap between 5367 and 5367-Vector in Fig. 6C.

Additionally, in Fig. 5A, the mock and vector panels for t24 appear to be very similar. The journal requested raw data and evidence of ethics approval, however the information provided by the Authors was not satisfactory. As a result, the Editors lost confidence in the integrity of this article’s findings.

Zhe Zhang disagrees with this retraction. Guojun Zhang, Chuize Kong, Bo Zhan, Xiao Dong and Xiaojun Man did not respond to the correspondence about this retraction.

